# Non-dispersive Fano resonances in hybrid plasmonic-distributed Bragg reflector structures

**DOI:** 10.1515/nanoph-2023-0054

**Published:** 2023-07-14

**Authors:** Shuangshuang Wang, Huatian Hu, Xiaoze Liu, Tao Ding

**Affiliations:** Key Laboratory of Artificial Micro- and Nano-structures of Ministry of Education of China, School of Physics and Technology, Wuhan University, Wuhan 430072, China; Hubei Key Laboratory of Optical Information and Pattern Recognition, Wuhan Institute of Technology, Wuhan 430205, China; Wuhan Institute of Quantum Technology, Wuhan 430206, China; Institute of Microscale Optoelectronics, Shenzhen University, Shenzhen, Guangdong, 518060, China

**Keywords:** angle dependence, distributed Bragg reflectors, Fano resonances, hybrid plasmon-dielectric structures, plasmonic

## Abstract

Fano resonance due to coupling of plasmon mode and Bragg modes is revealed without strong angular dependence based on Au nanoparticle on distributed Bragg reflectors (Au NPoDBRs). This Fano interference involves three-modes-coupling: the nanoparticle’s plasmon resonance, dispersive Bragg modes, and non-dispersive Bragg modes. It can be interpreted as a consequence of two processes: plasmonic coupling between dispersive Bragg modes and broad plasmon mode, and the strong coupling between narrowed plasmonic mode and non-dispersive Bragg mode. This Fano interference shows little dependence on the incidence angle but high tunability with the top-layer thickness, which is exploitable for novel nanophotonic devices with dispersion engineering.

## 1 Introduction

Fano resonance, named after Ugo Fano for his discovery in 1961 [1], is an asymmetric resonance resulting from the interference between a narrow discrete resonance and a continuum (or a broad spectrum). Recently, Fano resonance has been observed and developed in many dielectric and plasmonic structures [2–11], leading to a wide range of applications [12, 13], such as sensing [14–16], optical switching [17, 18], electro-optics [18, 19], waveguiding [20–24], slow light control and topological optics [25]. Intriguingly, in hybrid plasmon-dielectric structures, these applications of Fano resonance can be largely empowered by taking advantage of highly-confined fields of plasmonic broad resonances and sharp resonance linewidths of dielectric photonic structures [26–30]. For this purpose, the field distributions and the spectral shapes with narrow linewidths in hybrid photonic structures become the focuses of most previous studies [12, 13]. These focuses have established the key features of these Fano resonances, and provided guidelines for parameter engineering in hybrid structures. However, the angular dependence of single nanoparticle Fano resonance, strongly correlated with the energy versus momentum dispersions, is much less explored for fundamental physical pictures and extended applications.

In this paper, we investigate the angle independent dispersions for the Fano resonances in a hybrid plasmon-dielectric structure and demonstrate the intuitive physical picture behind them. This hybrid structure is exemplified with a gold nanoparticle on distributed Bragg reflector (Au NPoDBR) where the Au NPs possess significantly narrowed resonance linewidths with various configurations [26] and the distributed Bragg reflectors (DBRs) support optical Bragg modes with strong angular dependence. As such, it provides a clear and convenient way to look into the dispersions of Fano resonances in this hybrid structure. Through the study of the Fano resonances in Au NPoDBR, the underlying physical picture is profiled and provides valuable insights to the design of more flexible and versatile hybrid structures for photonic applications such as sensing, waveguiding and topological optics.

## 2 Results and discussion

**Figure 1:**
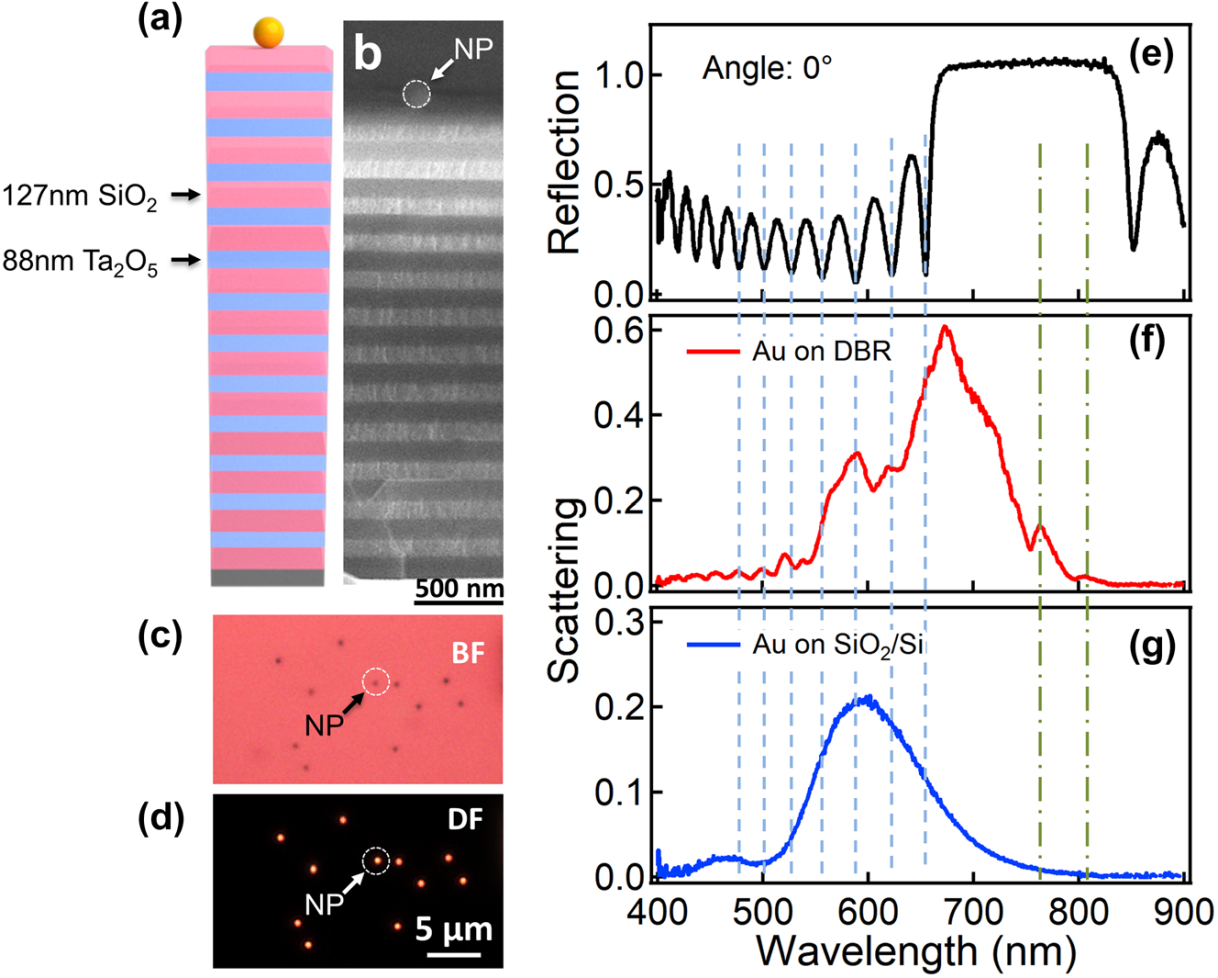
Fabrication of Au NPoDBR and optical characterizations. (a) Schematic of Au NPoDBR and (b) the corresponding SEM image of the cross section. (c) Bright-field and (d) dark-field optical images of Au NPoDBRs. (e) Reflection spectrum of DBR at normal incidence. Scattering spectra of 150 nm Au NPs on (f) DBR and (g) SiO_2_/Si substrate. The NA of the collection objective is 0.8.

The DBR substrates with 12 periods of Ta_2_O_5_/SiO_2_ layers were fabricated by ion-beam sputtering (Figure 1(a)). Each period is composed of 88 nm Ta_2_O_5_ and 127 nm SiO_2_. 150 nm Au NPs (BBI solution) were drop-casted on the DBR substrate to form the Au NPoDBR structure (Figure 1(b)). The Au NPs are well separated on the DBR substrate to avoid interparticle coupling, as seen in the bright field and dark field images (Figure 1(c) and (d), respectively). More details of the fabrication and characterization process can be found in Methods section. The reflection spectrum of the DBR shows a stopband centered at 750 nm (Figure 1(e) for normal incidence) with angular and polarization dependence (Supporting Information (SI), Figure S1). The scattering spectrum of the Au NPoDBRs (Figure 1(f)) appears to be quite different from those of Au on SiO_2_/Si substrate (Figure 1(g)). It manifests that fringed peaks are correlated with the Bragg modes outside the DBR stopband (especially at the blue side of stopband) at normal incidence in the way that the scattering peaks are closely corresponding to the Bragg dips (see the dashed lines in Figure 1(e) and (f)). As the Bragg modes are much narrower than the plasmonic mode of Au nanoparticles (NP) (Figure 1(g)), this close correspondence strongly suggests that the fringed peaks are the Fano resonance resulted from the plasmon mode and Bragg modes.

**Figure 2:**
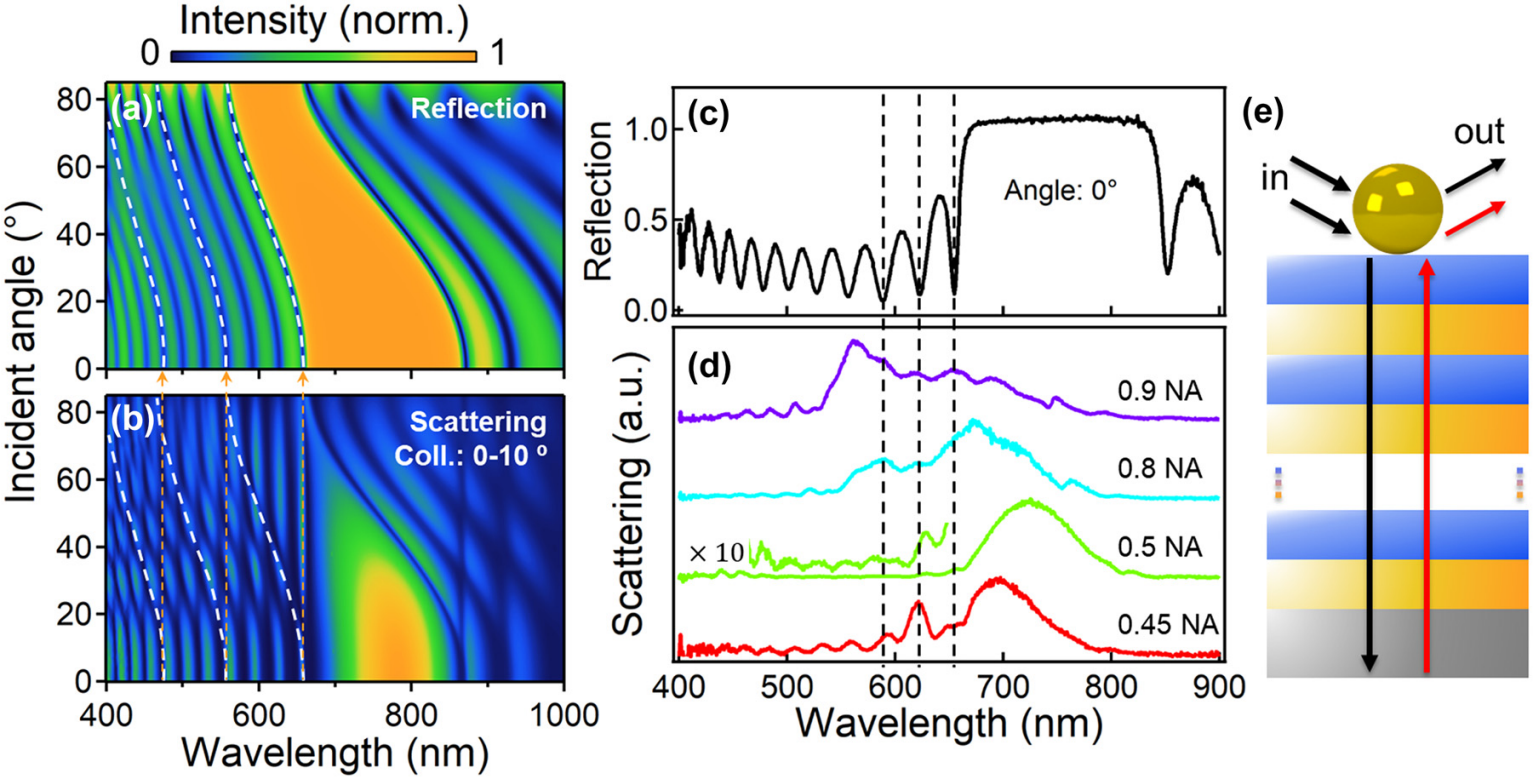
Angular dependent optical properties of Au NPoDBR and schematic illustration of Fano resonance. (a) Calculations of reflection spectra of DBR (orange dashed line represents the reflection at 0°) and (b) scattering spectra of Au NPoDBRs with increasing incidence (P-polarization) angle. The collection angle of the spectra ranges from 0° to 10°. (c) Experimental reflection spectra of DBR from normal incidence and (d) scattering spectra of Au NPoDBR collected with different NA of the objectives. (e) Schematic illustration of the physical processes of Fano resonance in the Au NPoDBR structure.

To confirm the Fano resonance from the DBR Bragg modes and the plasmon mode, angle-dependent reflection spectra of DBR (Figure 2(a)) and scattering spectra of Au NPoDBR (Figure 2(b)) are simulated. As the incidence angle increases, the Bragg modes blue-shift for the DBR reflection due to the increased contribution from the in-plane momentum (Figure 2(a)). Correspondingly, the scattering peaks for the Au NPoDBR slightly blue-shift with increasing angle, but show discontinuities with anti-crossing features (Figure 2(b)). With a closer look, this feature is resulted from the coupling between a non-dispersive mode (orange dashed lines) and angular dependent Bragg mode (white dashed lines). Here the “non-dispersive” refers to the shift of scattering dips which is almost negligible in comparison with the Bragg mode dispersion and linewidths.

In this context, we propose a physical picture of a spontaneous three-modes-coupling Fano resonance interplayed by the nanoparticle’s plasmon resonance, dispersive Bragg modes, and Bragg modes at normal incident (similar to a vertical cavity mode). This picture could be intuitively understood as the following two processes (Figure 2(e)): (1) The Au NP’s broad plasmonic resonance, considered as the super-radiant mode, [26] was filtered out by the dispersive Bragg modes. This process would give rise to the spectral feature for the dispersive scattering modes as Ref. [26], where the narrowed plasmonic linewidths and dispersion would be determined by the Bragg modes of DBR reflection. The coupling between DBR Bragg modes and plasmonic mode results in one-to-one correspondence between plasmonic scattering peaks and DBR reflection dips, acting like a “filtering” effect as discussed in Ref. [26]. (2) For the strongest reflectivity of Au NP at normal incidence, the reflected Bragg modes between Au NP and DBR act like vertical cavity modes, and thus show features of a non-dispersive discrete sub-radiant mode. These non-dispersive Bragg modes can strongly couple with the aforementioned hybridized mode, leading to the induced transparency features (dips). Although the Fano coupling is proved to be efficient for enhanced plasmonic sensing [26], the fundamental picture with the angular dependence is different. The first process of the angular dependence could be further substantiated and elaborated by the scattering spectra with larger collection angular range. On the contrary, the simulated modes by the coupling between regular Bragg modes and non-dispersive reflected ones become weak as the collection angle increases (SI Figure S2). As a result, the anti-crossing feature fades out with only the scattering of uncoupled reflected Bragg modes at larger angles. Indeed, in the experiment, we observe the fringed scattering peaks agree more with the normal incidence of Bragg modes at larger NA (larger collection angle: 0.9 NA, 0.8 NA), but show some slight deviation from those at smaller NA (0.5 NA, 0.45 NA) for the anti-crossing features (Figure 2(c) and (d)), which is also confirmed by the simulations (SI Figure S3). Both S- and P-polarization contribute to the scattering of Au NPoDBR with major from S-polarization while the P-polarization counts for the smaller shoulders (SI Figure S4). Furthermore, the temporal coupled-mode formalism [31] is carried out to describe a pair of interacting oscillators (with coupling strength *g*, vertical cavity total decay rate *γ*_0_, frequency *ω*_0_, and dispersive Bragg modes—*γ*_*v*_, *ω*_*v*_) coupled to a port and a bath via radiative (*γ*_*v* *r*_, *γ*_0*r*_) and nonradiative (*γ*_*vn*_, *γ*_0*n*_) decay channels, respectively [31].

(1) $$Q_{\text{sca}}=\gamma_{vr}^{2}{\left|i\left(\omega +\omega_{v}\right)+\gamma_{v}+\frac{g^{2}}{i\left(\omega -\omega_{0}\right)+\gamma_{0}}\right|}^{-2}+\text{BG}$$

where *γ*_*v*_ = *γ*_*vr*_ + *γ*_*vn*_, *γ*_0_ = *γ*_0*r*_ + *γ*_0*n*_, and a constant background (BG) is added to get a better fitting. According to the spectra fitting of both simulation and experiment (SI Figure S5), the coupling strength g between vertical cavity mode and dispersive Bragg modes is about 8 meV, manifesting itself as Fano interference in weak coupling regime.

**Figure 3:**
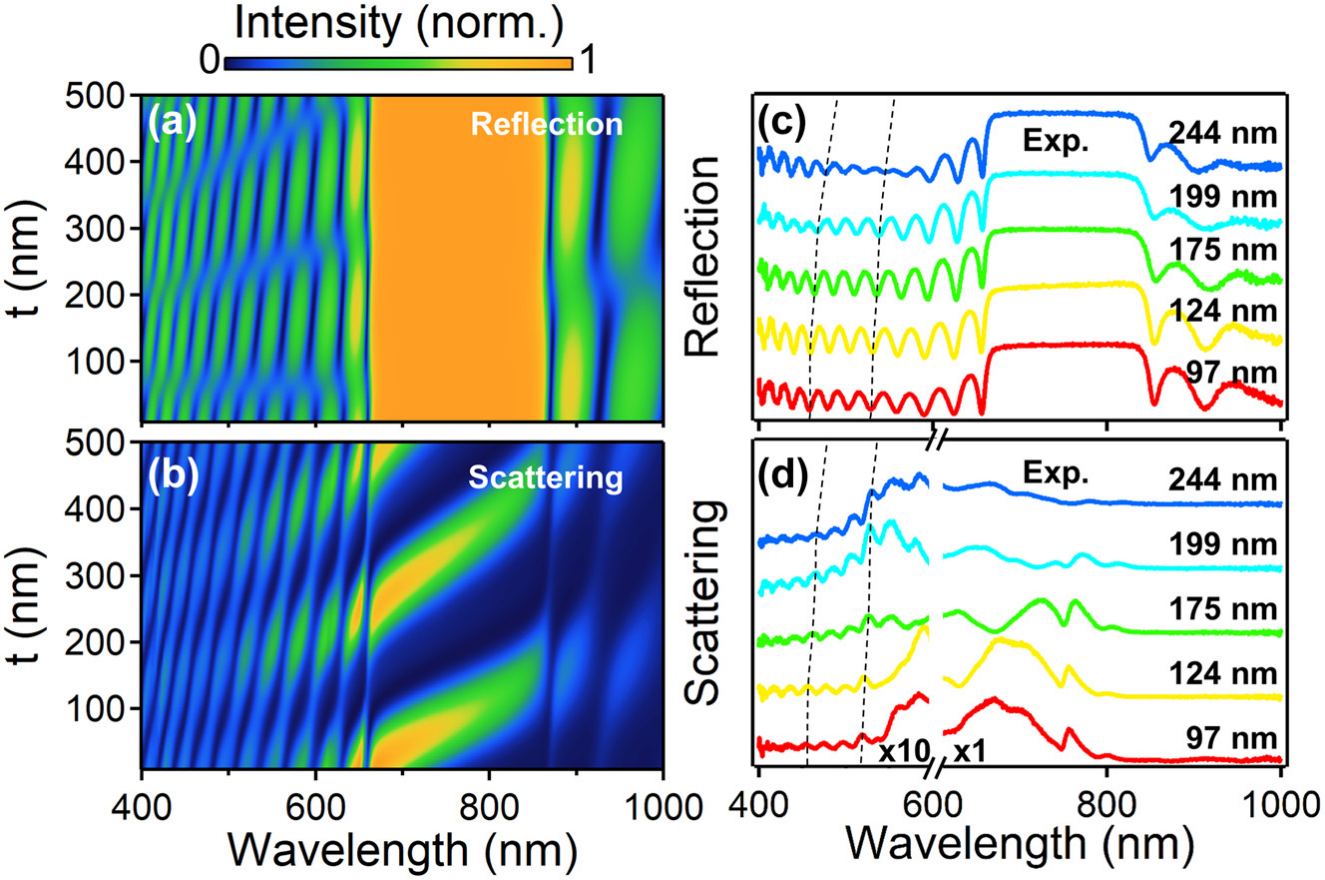
Tunable Fano interferences of Au NPoDBR by the top-layer SiO_2_ thickness. (a) and (c) Reflection spectra of DBR substrate with different thicknesses of top layer SiO_2_. (b) and (d) Scattering spectra of NPoDBR with different thicknesses of top layer SiO_2_. (a) and (b) are simulations and (c) and (d) are experimental results. The dashed curves are the guided lines for some of the coupled modes.

Though the scattering peaks show little dependence on the incidence angle, they can be flexibly tuned by the structural parameter, i.e., the thickness of top-layer SiO_2_. In Figure 3(a) and (b), the simulated reflectance and scattering spectra both red-shift with the increase of SiO_2_ thickness, which is further confirmed by the experimental data (Figure 3(c) and (d)). This is because the Fano coupling between the plasmon mode and the Bragg modes is largely tuned by the SiO_2_ thickness. Therefore, the tunability of this Fano resonance is quite sensitive to the structure parameters for the coupling process.

## 3 Conclusions

In summary, we have investigated the Fano resonance in hybrid Au NPoDBR structure. The scattering spectra of Au NPs were dramatically modified by the DBR substrate due to the Fano interference based on three modes: broad plasmon resonance of Au NP, dispersive DBR Bragg modes, and Bragg modes at normal incident. Based on such Fano interference, the scattering spectra show little angular dependence with almost angle independent dispersions. The investigation of such hybrid modes is vital for exploiting novel nanophotonic devices with strong angular and polarization dependence.

## 4 Methods

Twelve pairs of SiO_2_ and Ta_2_O_5_ DBR are deposited on Si substrate via ion beam sputtering (Veeco IBS) to achieve ultrahigh flatness and >99.95 % reflectivity. DBR substrate was then plasma cleaned, and monodispersed colloidal nanoparticles were drop-casted on top of it. The samples were put under a home-built microscope (Olympus BX53) for observation. Bright-/dark-field images of the samples were captured with a CCD camera (Infinity 3.0) and the scattering spectra of the nanoparticles were collected with different objectives (0.45NA, 0.5NA, 0.8NA, 0.95NA), which were confocally recorded through an optical fibre (dia. 50 μm) connected to a spectrometer (QEPro, Ocean Optics). The fiber aperture limits the spectra collection region within ∼1 μm. As the AuNPs are well separated with a distance of at least 10 μm, the collected signal of scattering through the fiber aperture is only from single Au NP. The angle resolved reflection spectra were collected using angle-resolved microscope (ARMS) equipment (FuXiang) in the visible wavelength range with 400 ms of integration. SEM images of the samples were captured with ZEISS SIGMA at the accelerating voltage of 5 kV.

Full-wave electromagnetic simulations were performed by finite element method (FEM). For the sake of computational simplicity, 2D model was established for the Au NPoDBR structure: 12 pairs of SiO_2_ (127 nm)/Ta_2_O_5_ (88 nm) layers were placed on a silicon substrate. An extra layer of SiO_2_ was added on the top. Au NPs was placed on the top of the SiO_2_ layer. The refractive indices of SiO_2_ and Ta_2_O_5_ were 1.48 and 2.13, respectively. The permittivity of Au and silicon followed the work by Johnson [32], and Green [33], respectively. For calculating the reflection spectra of DBR (without nanoparticle), one-step model was utilized where the plane wave was introduced by ports. Floquent periodic boundary conditions were applied. As for calculating the scattering spectra from the Au NPs, a two-step model was established which took the aforementioned reflection field of DBR as a background to separate the scattering from the nanoparticle. The whole structure was covered by perfect matching layers. The scattering was calculated with surface integral of the scattered-field Poynting vector with collection NA of 0.8. A near-to-far-field transformation was applied using open-source code from Yang et al. [34].

## Supplementary Material

Supplementary Material Details
