# Supplementary material for: Non-dispersive Fano resonances in hybrid plasmonic-distributed Bragg reflector structures
Source: Nanophotonics. 2023 Jul 14;12(16):3211–6. doi: 10.1515/nanoph-2023-0054 (PMC11501441; doi:10.1515/nanoph-2023-0054)
Supplement: Supplementary file 1 — Supplementary Material Details [file j_nanoph-2023-0054_suppl_001.docx]

**Supporting Information for**

Non-dispersive Fano Resonances in hybrid plasmonic-distributed Bragg reflector structures

Shuangshuang Wang^1,4†^, Huatian Hu^2,†^, Xiaoze Liu^1,3*^, Tao Ding^1,*^

1 Key Laboratory of Artificial Micro- and Nano-structures of Ministry of Education of China, School of Physics and Technology, Wuhan University, Wuhan 430072, China

2 Hubei Key Laboratory of Optical Information and Pattern Recognition, Wuhan Institute of Technology, Wuhan 430205, China

3 Wuhan Institute of Quantum Technology, Wuhan 430206, China

4 Institute of Microscale Optoelectronics, Shenzhen University, Shenzhen, Guangdong, 518060, China

^†^ equal contributions

This document provides the supporting information for the main manuscript.


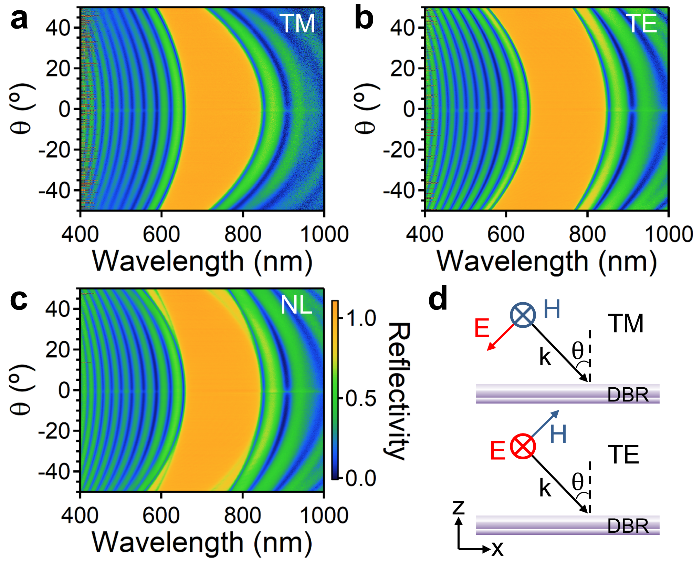


**Figure S1. Angle-resolved reflection spectra detected with different polarizations.** Reflection spectra of DBR with different angles of incidence. (a) TM (P-Polarization), (b) TE (S-Polarization), (c) natural light (NL, unpolarized) respectively. (d) Schematic illustration of the TM/TE polarized incidence on the DBR.


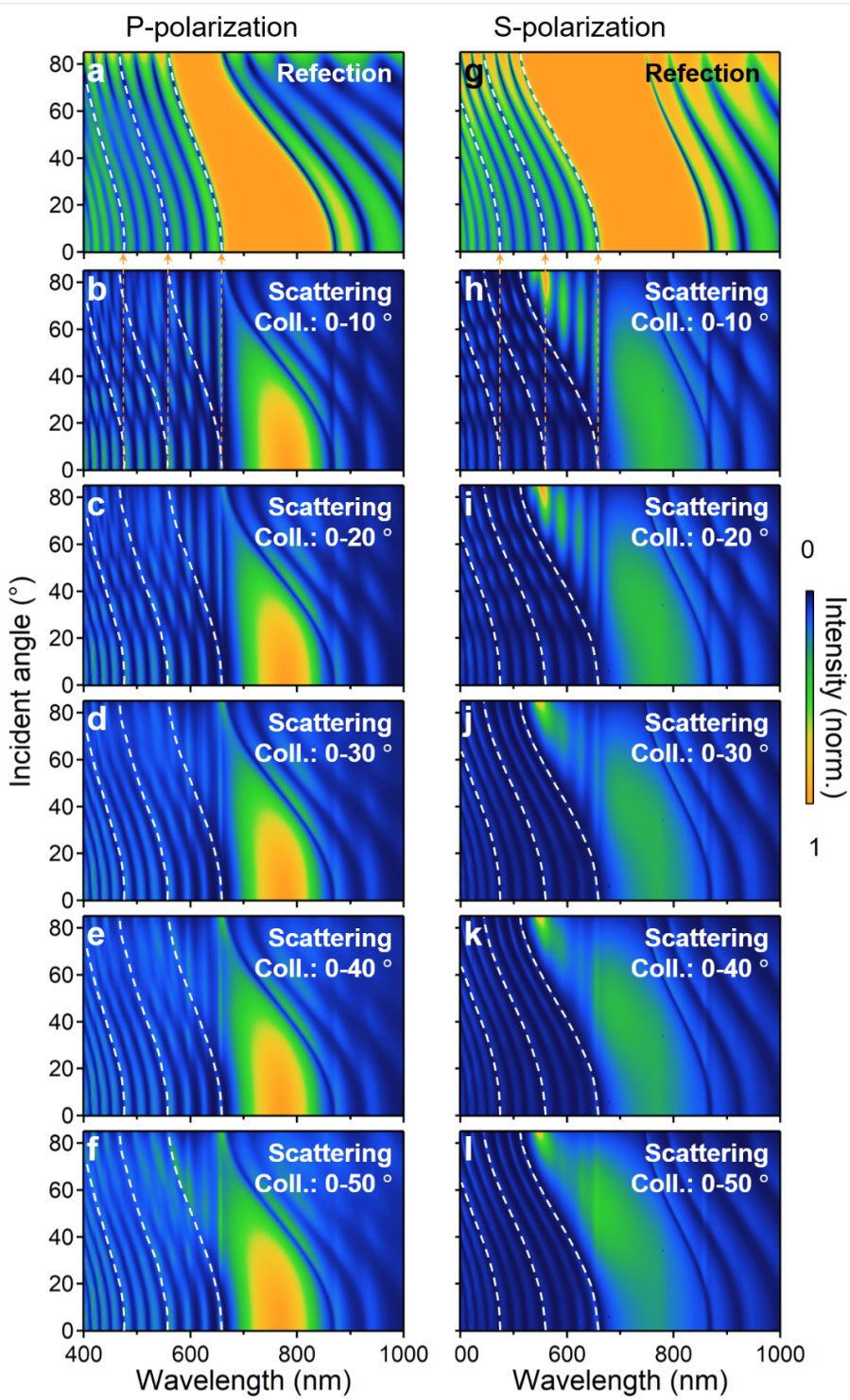


**Figure S2. Simulated reflection and scattering spectra of Au NPoDBRs with different angles of incidence.** (a, g) Calculated reflection spectra of DBR and (b-l) scattering spectra of Au NPoDBRs with increasing angle of incidence. The collection angle of the scattering spectra ranges from $0-10^{\circ}$(b, h), $0-20^{\circ}$(c, i), $0-30^{\circ}$(d, j), $0-40^{\circ}$(e, k), $0-50^{\circ}$(f, l) and the modes by the coupling between regular Bragg modes and non-dispersive reflected ones become weak as the collection angles increase for both P-poarization (a-f) and S-polarization (g-l) excitation. The white dashed lines represent DBR reflection dip modes change with angle and orange dashed line indicate the cavity mode of DBR (normal incident).


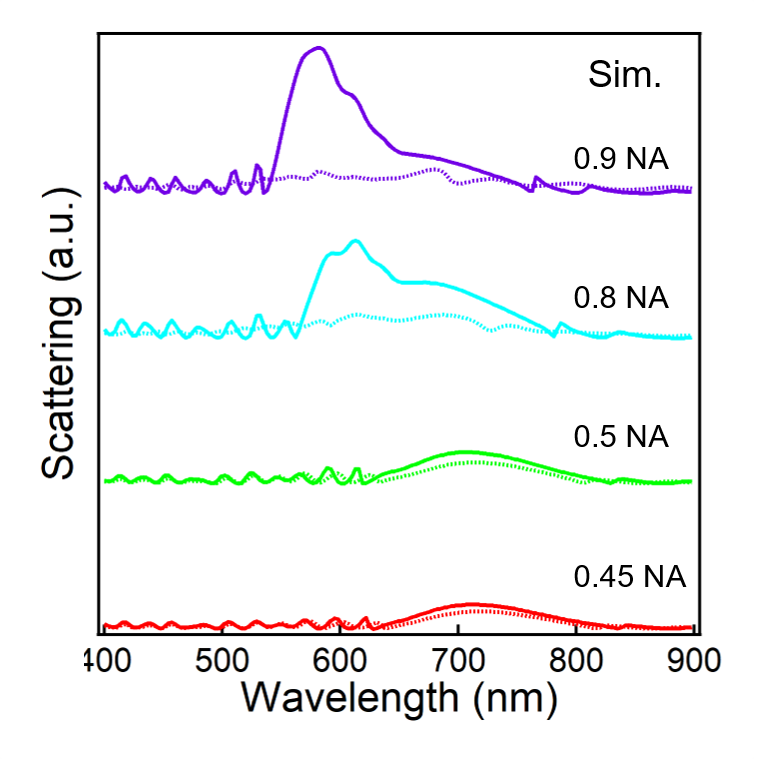


**Figure S3. Simulated scattering spectra of Au NPoDBR collected with different NA of objectives.** Solid lines and dashed lines represent S- and P-polarization excitation respectively.


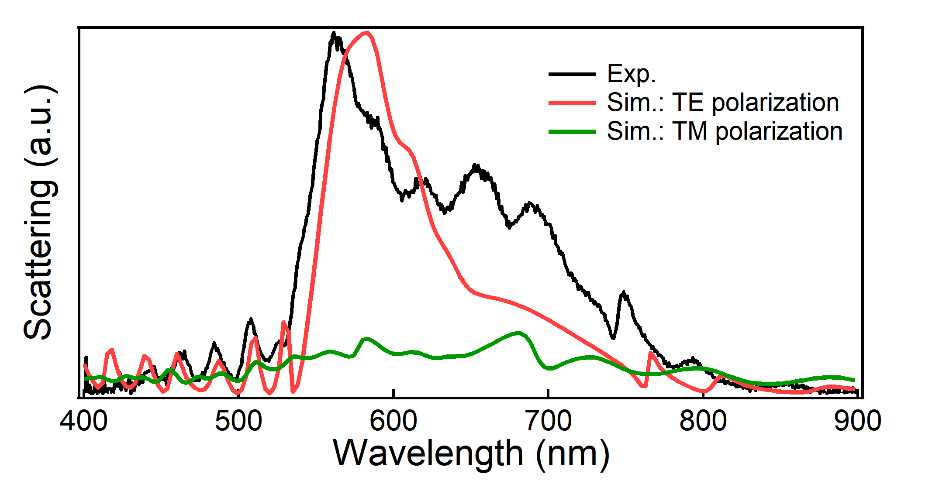


**Figure S4. Scattering spectra of Au NPoDBR collected with objective of 0.9 NA (black line) and the corresponding simulated scattering spectra with excitation of TE (S-polarization) and TM (P-polarization) light.** Both TE and TM polarization contribute to the experimental scattering. Deviations between simulations and experiments could be attributed to some small discrepancies of the material constants and geometry setup of the models from real samples.


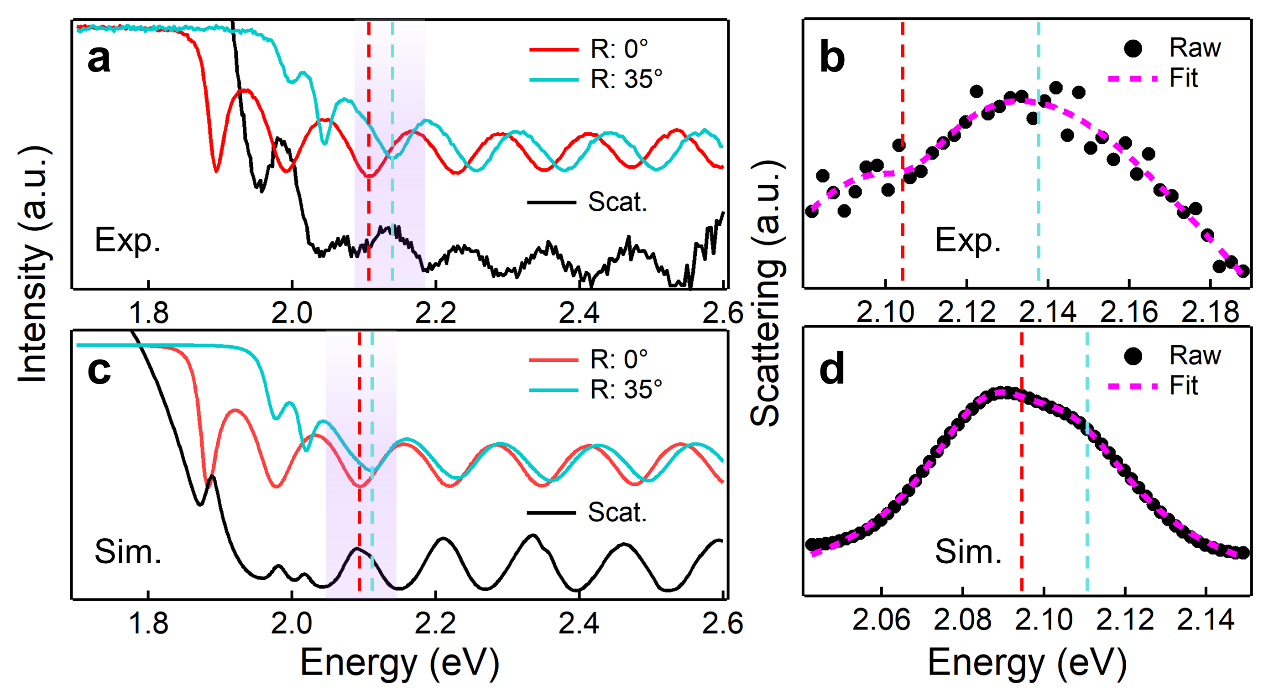


**Figure S5. Sprectral fitting via temporal coupled-mode formalism.** (a, b) Experiments and (c, d) Simulations. Vertical cavity modes (red dashed lines) appeared in the reflection spectra from normal incidence and dispersive Bragg modes (bule dashed lines) appeared in reflection spectra from angled incidence (35°) couple together which induce transparency features. The fitting parameters for experiments (a, b) are $\gamma_{vr}=0.03322$ eV, $\gamma_{vn}=0.06231$ eV, $\gamma_{0}=0.01361$ eV, $\omega_{v}=2.12701$ eV, $\omega_{0}=2.10549$ eV and $g=0.00767$ eV, and for simulations (c, d) are $\gamma_{vr}=0.00714$ eV, $\gamma_{vn}=0.01528$ eV, $\gamma_{0}=0.0175$ eV, $\omega_{v}=2.09503$ eV, $\omega_{0}=2.09801$ eV and $g=0.00818$ eV. Deviations between simulations and experiments is mainly due to some small discrepancies of the material constants and geometry setup of models from the real samples.
